# Knowledge, attitudes, and practices among users of GLP-1 receptor agonists in Saudi Arabia: a cross-sectional study

**DOI:** 10.3389/fphar.2026.1912202

**Published:** 2026-10-12

**Authors:** Abdullah A. Alshehri, Mohammed K. Al-Osaimi, Nawaf N. Al-Saeedi, Osama M. Al-Wagdani, Sultan S. Al-Zahrani, Fareed A. Alharthi, Ghadah H. Alshehri, Fahad H. Baali, Saeed Saad A. Alghamdi, Wael Y. Khawagi

**Affiliations:** 1 Department of Clinical Pharmacy, College of Pharmacy, Taif University, Taif, Saudi Arabia; 2 College of Pharmacy, Taif University, Taif, Saudi Arabia; 3 Medical Center, Facilities Security Forces Training Center, Jeddah, Saudi Arabia; 4 Department of Pharmacy Practice, College of Pharmacy, Princess Nourah bint Abdulrahman University, Riyadh, Saudi Arabia

**Keywords:** GLP-1 receptor agonists, health literacy, knowledge attitudes practices, medication safety, Saudi Arabia, semaglutide, tirzepatide, weight-loss medications

## Abstract

**Background:**

The use of glucagon-like peptide-1 (GLP-1) receptor agonists has increased rapidly in Saudi Arabia for weight management and glycaemic control. However, concerns remain regarding inappropriate use, medication safety, and misconceptions surrounding these therapies. This study assessed the knowledge, attitudes, and practices (KAP) of GLP-1 receptor agonist users in Saudi Arabia and identified factors associated with these domains.

**Methods:**

A cross-sectional study was conducted among adult GLP-1 receptor agonist users in Saudi Arabia between December 2025 and March 2026. Eligible participants were aged ≥18 years and had used semaglutide, liraglutide, dulaglutide, or tirzepatide within the previous 12 months. Participants were recruited through purposive snowball sampling via social media platforms. Descriptive statistics and binary logistic regression were used to assess KAP levels and associated factors.

**Results:**

A total of 310 participants were included. The median age was 33 years (IQR: 16), and the median BMI was 33.9 kg/m^2^ (IQR: 7.1). Tirzepatide was the most frequently used medication (46.1%). Good practices were reported by 54.8% of participants, whereas only 25.8% demonstrated good knowledge and 33.2% reported positive attitudes. Knowledge, attitude, and practice scores were positively correlated (ρ = 0.278–0.395; all p < 0.001). Participants aged >50 years were less likely to demonstrate good knowledge (aOR = 0.14, 95% CI: 0.04–0.82) and positive attitudes (aOR = 0.29, 95% CI: 0.09–0.91). Postgraduate education was independently associated with positive attitudes (aOR = 6.51, 95% CI: 2.23–18.99) and good practices (aOR = 4.47, 95% CI: 1.37–14.60), while female sex was associated with good practices (aOR = 2.06, 95% CI: 1.28–3.33).

**Conclusion:**

GLP-1 receptor agonist users demonstrated generally favourable medication-use practices but suboptimal knowledge and attitudes. Higher knowledge was associated with more positive attitudes and better practices, highlighting the importance of patient education and healthcare professional counselling to support safe and informed use.

## Introduction

Obesity is one of the leading global public health challenges, contributing substantially to morbidity, mortality, reduced quality of life, and increasing healthcare expenditure worldwide (Brauer et al., 2024; WHO, 2025). More than one billion individuals are living with obesity, with prevalence rates continuing to rise across both developed and developing countries (WHO, 2025; Phelps et al., 2024). In Saudi Arabia, obesity and overweight represent major health concerns driven by rapid urbanisation, sedentary lifestyles, unhealthy dietary habits, and reduced physical activity (Salem et al., 2022; DeNicola et al., 2015). Current national estimates indicate that obesity affects a substantial proportion of the adult population and is strongly associated with chronic conditions such as type 2 diabetes mellitus, hypertension, dyslipidaemia, and cardiovascular disease (Alshahrani et al., 2026). Consequently, obesity imposes a substantial clinical and economic burden on the healthcare system and highlights the urgent need for effective long-term management strategies (Wolfenden et al., 2019; Fruh, 2017).

In recent years, glucagon-like peptide-1 (GLP-1) receptor agonists and dual glucose-dependent insulinotropic polypeptide (GIP)/GLP-1 receptor agonists have emerged as important pharmacological therapies for obesity and diabetes management (Nauck et al., 2026; Liu, 2024). Medications such as semaglutide, liraglutide, dulaglutide, and tirzepatide have demonstrated clinically significant reductions in body weight and improvements in glycaemic control in large randomised controlled trials (Aamir et al., 2025; Harbi et al., 2026). These medications exert their therapeutic effects through multiple mechanisms, including delayed gastric emptying, appetite suppression, enhanced satiety, and improved insulin secretion (Nauck et al., 2026; Liu, 2024). Their increasing availability and expanding clinical indications have contributed to widespread use globally and within Saudi Arabia.

While GLP-1 receptor agonists have demonstrated substantial efficacy in weight reduction and glycaemic control, concerns regarding their safe and appropriate use have increasingly emerged (Moyad, 2023). These medications are associated with several adverse effects and require appropriate clinical monitoring and patient counselling to ensure safe and effective use. Common adverse effects include nausea, vomiting, diarrhoea, and gastrointestinal discomfort, whereas more serious complications such as pancreatitis, gallbladder disease, dehydration, and potential drug interactions have also been reported (Sodhi et al., 2023). In Saudi Arabia, the growing popularity of GLP-1 receptor agonists has coincided with increasing public awareness and substantial interest in their use for weight management, with social media representing an important source of information about these therapies (Abualhommos et al., 2026; Zalat et al., 2025; Alfares et al., 2025). However, concerns remain regarding inappropriate use, unrealistic expectations, inadequate medical supervision, and misconceptions that these medications can replace dietary modification and physical activity (Wharton et al., 2020; Maqsood et al., 2026). Reports from different settings have highlighted issues related to self-medication, off-label use for cosmetic purposes, obtaining medications without appropriate clinical assessment, and discontinuation without professional follow-up, all of which may compromise patient safety and treatment effectiveness.

Knowledge, attitudes, and practices (KAP) studies are widely used to evaluate public understanding, perceptions, and behaviours related to medication use and health interventions (Antwi et al., 2020; Alruqayb et al., 2025; Vargas-Garrido, 2026). Despite the rapidly increasing use of GLP-1 receptor agonists in Saudi Arabia, evidence regarding users’ KAP toward these medications remains limited. Previous research has primarily focused on awareness and attitudes within the general population or awareness among users within limited geographic settings, rather than providing a comprehensive national assessment of knowledge, attitudes, and real-world medication-use practices among users of GLP-1 receptor agonist (Abualhommos et al., 2026; Al saleh et al., 2020). Understanding how individuals perceive and use these therapies is essential for improving patient safety, optimising treatment outcomes, and supporting rational medication use. Therefore, this study aimed to assess the knowledge, attitudes, and practices of GLP-1 receptor agonist users in Saudi Arabia and to identify sociodemographic and clinical factors associated with these domains. The findings may help inform targeted educational initiatives, pharmacy counselling strategies, and public health policies related to obesity pharmacotherapy in the Kingdom.

## Methods

### Study design and participants

A cross-sectional study was conducted using an online self-administered questionnaire among adult users of GLP-1 receptor agonists in Saudi Arabia between December 2025 and March 2026. Eligible participants were adults aged ≥18 years residing in Saudi Arabia who had personally used semaglutide (Ozempic®, Wegovy®), liraglutide (Saxenda®, Victoza®), dulaglutide (Trulicity®), or tirzepatide (Mounjaro®) within the preceding 12 months. Individuals who had never used GLP-1 receptor agonists or submitted incomplete responses were excluded from the study.

### Questionnaire development

The questionnaire was developed after a comprehensive review of relevant literature and by adapting items from previously validated KAP instruments to suit the Saudi context agonist (Abualhommos et al., 2026; Al saleh et al., 2020). The final questionnaire comprised four sections: sociodemographic and clinical characteristics (11 items), knowledge (8 items; Yes/No/Don’t know), attitudes (9 items; five-point Likert), and practices (9 items; Always/Sometimes/Never). The knowledge domain assessed participants’ understanding of therapeutic indications, dosing requirements, storage conditions, mechanisms of action, and potential adverse effects. The attitude domain explored perceptions regarding safety, effectiveness, necessity of medical supervision, and willingness to adopt or continue therapy. The Practice domain evaluated behaviors related to healthcare consultation, medication adherence, dose titration, and methods of obtaining medications. Knowledge items were scored as one for correct and 0 for incorrect or “Don’t know” responses (total score: 0–8) and categorized as poor (0–2), average (3–5), or good (6–8). Attitude items were scored on a five-point Likert scale (total score: 9–45) and categorized as negative (9–15), neutral (16–30), or positive (31–45). Practice items yielded a total score of 0–18 and were categorized as poor (0–6), moderate (7–12), or good (13–18). These cutoffs were predefined for analytical purposes and were not considered externally validated clinical thresholds.

The questionnaire was initially developed in English and subsequently translated into Arabic using a forward–backward translation process to ensure linguistic accuracy and conceptual equivalence. The Arabic version was used for data collection to enhance clarity, accessibility, and cultural appropriateness for the target population. Face and content validity were evaluated by three academic experts in clinical pharmacy and epidemiology. A pilot study involving 14 individuals representative of the target population was conducted to assess the clarity, comprehensibility, cultural appropriateness, and acceptability of the questionnaire. Minor revisions were made based on participant feedback, and pilot data were excluded from the final analysis. Internal consistency reliability of the questionnaire was assessed using Cronbach’s alpha coefficient. The overall Cronbach’s alpha value was 0.846, indicating good internal consistency and reliability of the study instrument. A Cronbach’s alpha coefficient of ≥0.70 was considered indicative of acceptable reliability.

### Sample size calculation

The minimum required sample size was calculated using Raosoft® website, assuming a 95% confidence level, 5% margin of error, and 50% response distribution, yielding a minimum target of 385 participants.

### Data collection and recruitment

A structured questionnaire administered through Google Forms. The survey link was distributed via commonly used social media and communication platforms in Saudi Arabia, including WhatsApp and Telegram, employing a purposive snowball sampling approach. Before accessing the questionnaire, participants were presented with an introductory statement explaining the study objectives, eligibility criteria, voluntary nature of participation, and confidentiality assurances. Electronic informed consent was obtained from all participants prior to questionnaire completion.

### Statistical analysis

Descriptive statistics were used to summarize the study data. Categorical variables were presented as frequencies and percentages, whereas continuous variables were expressed as means and standard deviations or medians and interquartile ranges (IQRs), as appropriate. Normality of continuous variables was assessed using the Shapiro–Wilk test. Since the knowledge, attitude, and practice (KAP) scores were not normally distributed (Shapiro–Wilk test, p < 0.001), these variables were summarized using medians and IQRs.

Associations between categorical variables were evaluated using the chi-square test. Spearman’s rank correlation analysis was performed to assess correlations among total knowledge, attitude, and practice scores. Comparisons of KAP scores across sociodemographic groups were conducted using the Wilcoxon rank-sum test for variables with two categories and the Kruskal–Wallis test for variables with three or more categories.

Binary logistic regression analysis was performed to identify factors independently associated with high knowledge, positive attitude, and good practice levels. Crude odds ratios (CORs) and adjusted odds ratios (aORs) with 95% confidence intervals (CIs) were reported. Variables with p < 0.20 in univariate analysis were included in the multivariable logistic regression models. Model goodness-of-fit was assessed using the Hosmer–Lemeshow test, which demonstrated adequate model fit for the knowledge, attitude, and practice models (p = 0.136, 0.756, and 0.998, respectively). A two-sided p-value <0.05 was considered statistically significant. All analyses were performed using RStudio software (version 4.4.1).

### Ethical considerations

Ethical approval was obtained from the Research Ethics Committee of Taif University (Approval No. 47097; 7 December 2025). The study was conducted in accordance with the principles of the Declaration of Helsinki. No personal identifiers were collected, and all data were treated confidentially and used exclusively for research purposes.

## Results

### Characteristics of participants

A total of 310 participants were included in the study. Females constituted 56.8% of the sample, and the median age was 33 years (IQR: 16). More than half of the participants held a bachelor’s degree (56.5%). The highest proportions of participants were from Makkah (31.3%) and Riyadh (25.8%) regions, followed by the Eastern Province (10.3%). Most participants reported no chronic disease (61.6%), while type 2 diabetes mellitus was the most common comorbidity (20.0%). Only 8.7% of participants identified as healthcare practitioners. The median body mass index (BMI) was 33.87 kg/m^2^ (IQR: 7.13), with the majority of participants classified as having obesity (79.4%). Regarding GLP-1 receptor agonist use, tirzepatide was the most frequently reported medication (46.1%), followed by semaglutide (34.2%) and Liraglutide (9.4%) (Table 1).

**TABLE 1:** Characteristics of participants using GLP-1 receptor agonists.

Characteristics	n (%)
Gender	
Male	134 (43.2)
Female	176 (56.8)
Age (years)	
Median (IQR)	33 (16)
≤30	129 (41.6)
31–40	88 (28.4)
41–50	61 (19.7)
>50	32 (10.3)
Education level	
High school or less	77 (24.8)
Diploma	34 (11.0)
Bachelor’s degree	175 (56.5)
Postgraduate studies	24 (7.7)
Region of residence	
Makkah	97 (31.3)
Riyadh	80 (25.8)
Eastern Province	32 (10.3)
Al-Madinah	23 (7.4)
Al-Qassim	23 (7.4)
Other regions	55 (17.7)
Chronic disease	
None	191 (61.6)
Type 2 diabetes mellitus	62 (20.0)
Hypertension	16 (5.2)
Hyperlipidaemia	9 (2.9)
Other chronic diseases	32 (10.3)
Healthcare practitioner	
Yes	27 (8.7)
No	283 (91.3)
Body mass index (BMI) kg/m^2^	
Median (IQR)	33.87 (7.13)
Normal weight (<25 kg/m^2^)	9 (2.9)
Overweight (25–29.9 kg/m^2^)	55 (17.7)
Obesity (≥30 kg/m^2^)	246 (79.4)
GLP-1 receptor agonist used	
Tirzepatide	143 (46.1)
Semaglutide (Ozempic®)	106 (34.2)
Liraglutide (Saxenda®)	29 (9.4)
Semaglutide (Wegovy®)	10 (3.2)
Liraglutide (Victoza®)	8 (2.6)
Multiple medications	14 (4.5)

### Levels of knowledge, attitudes, and practices toward GLP-1 receptor agonist

Most participants demonstrated moderate knowledge regarding GLP-1 receptor agonists (61.9%), whereas 25.8% exhibited good knowledge and 12.3% had poor knowledge. Similarly, the majority reported a neutral attitude (66.1%), while 33.2% demonstrated a positive attitude. Practice outcomes were generally more favorable, with 54.8% of participants demonstrating good practice, compared with 42.9% exhibiting moderate practice and 2.3% poor practice. The distribution of participants across KAP categories is presented in Figure 1. Detailed responses to individual knowledge, attitude, and practice items are presented in Supplementary Tables S1–S3.

**FIGURE 1:**
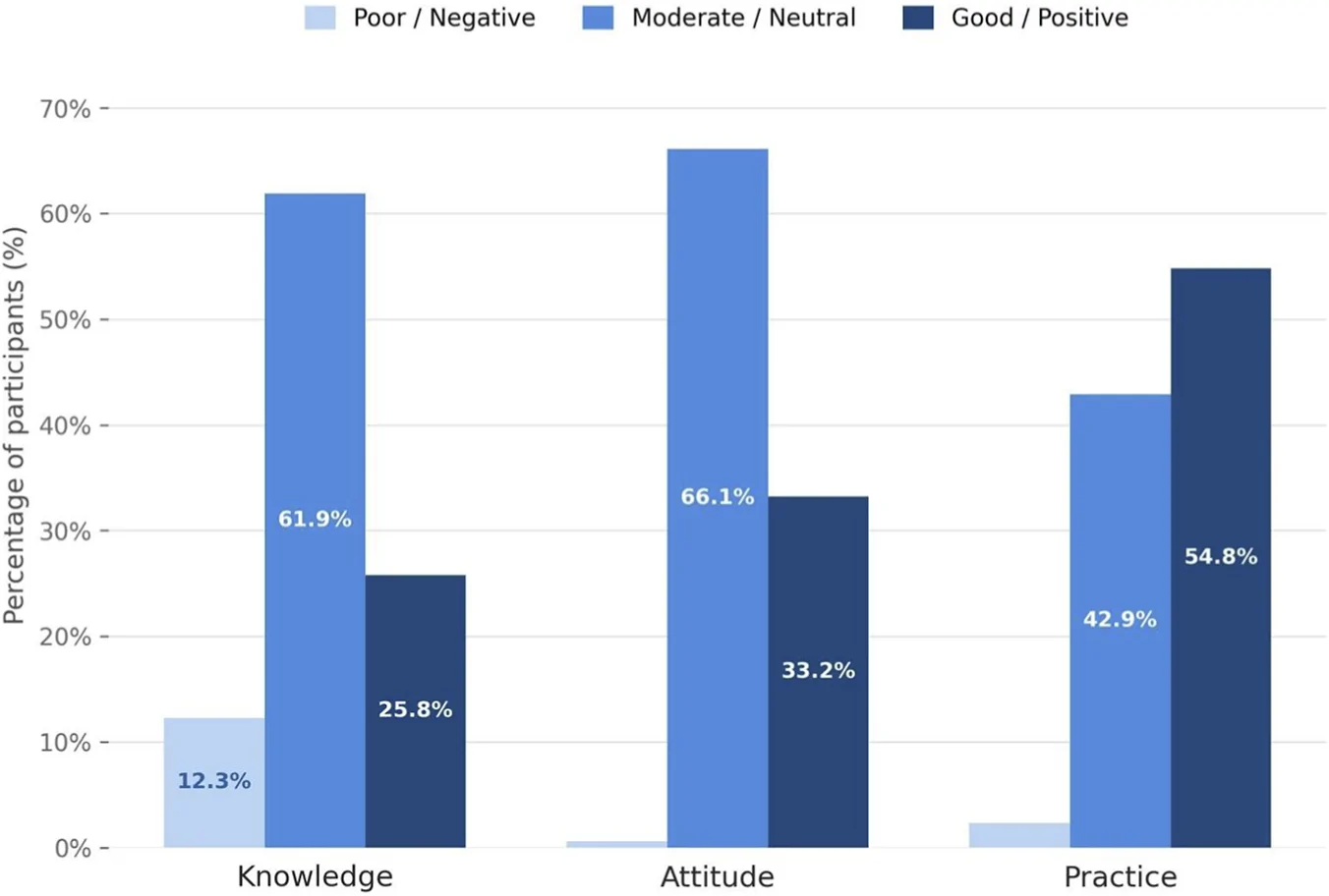
Distribution of participants according to knowledge, attitude, and practice levels regarding GLP-1 receptor agonist.

### Associations between knowledge, attitude, and practice levels

Significant positive correlations were observed among all KAP domains. Knowledge was moderately correlated with attitude (ρ = 0.375, p < 0.001) and weakly correlated with practice (ρ = 0.278, p < 0.001). Attitude also demonstrated a moderate positive correlation with practice (ρ = 0.395, p < 0.001). Scatter plots further illustrated the positive relationships between the KAP domains (Figure 2).

**FIGURE 2:**
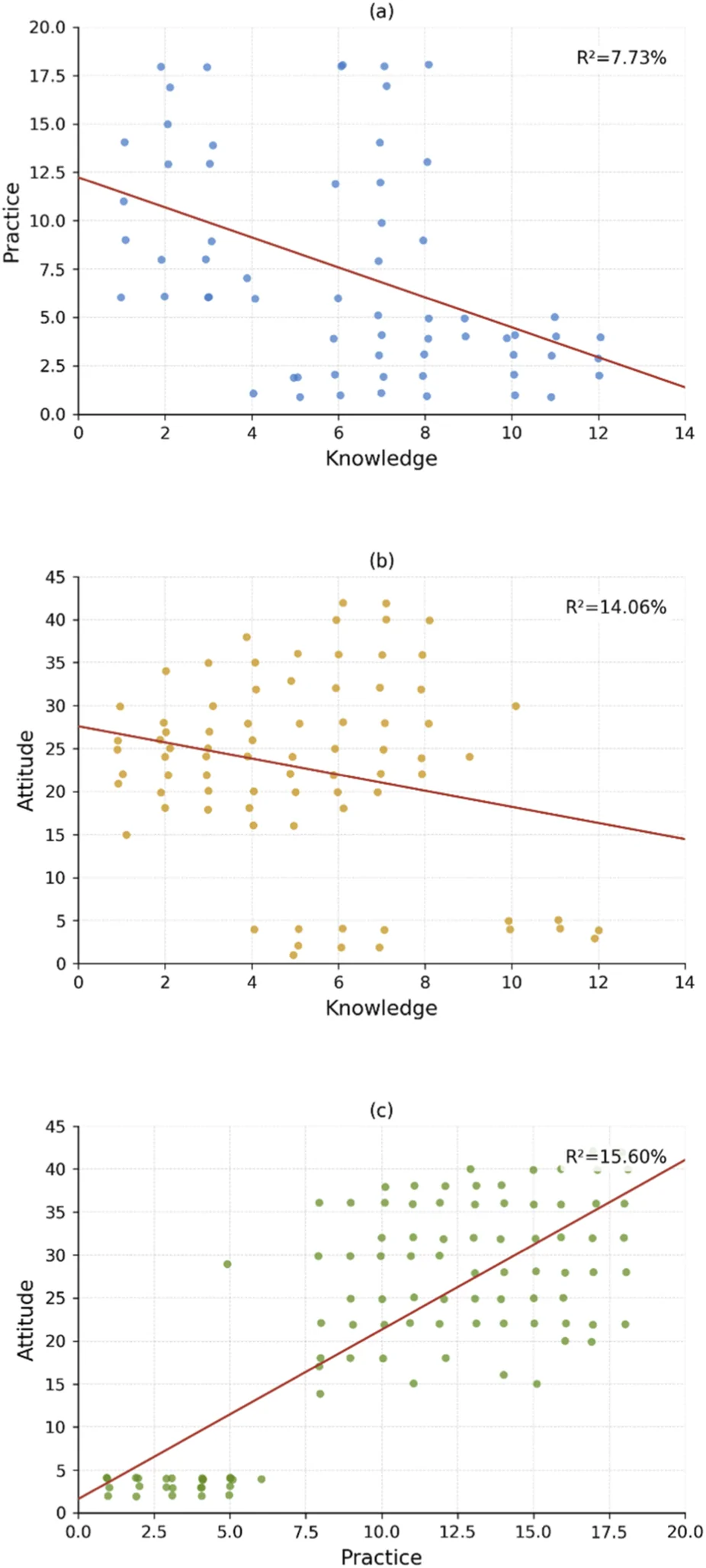
Scatter plots showing correlations between KAP scores: **(a)** Practice vs. Knowledge, **(b)** Attitude vs. Knowledge, and **(c)** Attitude vs. Practice.

Significant associations were observed among all KAP domains. Participants with good knowledge were more likely to demonstrate positive attitudes (63.7%) and good practices (72.5%) than participants with poor knowledge (both p < 0.001). Similarly, participants with positive attitudes were more likely to exhibit good practices (75.7%) than those with neutral attitudes (44.4%) (Table 2).

**TABLE 2:** Associations between knowledge, attitude, and practice categories among participants.

Attitude distribution by knowledge category	Negative attitude	Neutral attitude	Positive attitude	p-value
Poor Knowledge	1 (2.6%)	32 (84.2%)	5 (13.2%)	<0.001
Average Knowledge	1 (0.5%)	144 (75.0%)	47 (32.7%)	
Good Knowledge	0 (0.0%)	29 (36.3%)	51 (63.7%)	

Practice distribution by knowledge category	Poor practice	Moderate practice	Good practice	p-value
Poor Knowledge	5 (13.2%)	20 (52.6%)	13 (34.2%)	<0.001
Average Knowledge	0 (0.0%)	93 (48.4%)	99 (51.6%)	
Good Knowledge	2 (2.5%)	20 (25%)	58 (72.5%)	

Practice distribution by attitude category	Poor practice	Moderate practice	Good practice	p-value
Negative Attitude	0 (0.0%)	1 (50%)	1 (50%)	<0.001
Neutral Attitude	7 (3.4%)	107 (52.2%)	91 (44.4%)	
Positive Attitude	0 (0.0%)	25 (24.3%)	78 (75.7%)	

### Factors associated with knowledge, attitudes, and practices

Multivariable logistic regression analyses identified several independent predictors of KAP outcomes. Participants aged >50 years were significantly less likely to demonstrate good knowledge (aOR = 0.14, 95% CI: 0.04–0.82) and positive attitudes (aOR = 0.29, 95% CI: 0.09–0.91) than those aged ≤30 years. Postgraduate education was independently associated with positive attitudes (aOR = 6.51, 95% CI: 2.23–18.99) and good practices (aOR = 4.47, 95% CI: 1.37–14.60). Female participants were more likely than males to demonstrate good practices (aOR = 2.06, 95% CI: 1.28–3.33). No significant independent associations were observed between the type of GLP-1 receptor agonist used and KAP outcomes after adjustment for potential confounders (Table 3). Detailed univariate regression analyses are provided in Supplementary Tables S4–S6.

**TABLE 3:** Multivariable logistic regression analysis of factors associated with knowledge, attitudes, and practices toward GLP-1 receptor agonist.

Variable	Good knowledge %	Good knowledge aOR (95% CI)	Positive attitude %	Positive attitude aOR (95% CI)	Good practice %	Good practice aOR (95% CI)
Age (years)						
≤30	28.7%	1	37.2%	1	58.1%	—
31–40	28.4%	0.88 (0.47–1.67)	42.0%	1.09 (0.60–1.99)	53.4%	—
41–50	26.2%	0.93 (0.45–1.94)	23.0%	0.52 (0.25–1.11)	49.2%	—
>50	6.3%	0.14 (0.04–0.82)	12.5%	0.29 (0.09–0.91)	56.3%	—
Gender						
Male	18.7%	1	24.6%	1	43.3%	1
Female	31.3%	1.71 (0.97–3.04)	39.8%	1.65 (0.96–2.82)	63.6%	2.06 (1.28–3.33)
Education level						
High school or less	22.1%	1	23.4%	1	46.8%	1
Diploma	11.8%	0.42 (0.13–1.43)	20.6%	0.82 (0.30–2.27)	52.9%	1.23 (0.54–2.84)
Bachelor’s degree	28.0%	1.15 (0.59–2.24)	34.9%	1.69 (0.89–3.21)	54.9%	1.35 (0.77–2.37)
Postgraduate studies	41.7%	1.95 (0.71–5.36)	70.8%	6.51 (2.23–18.99)	83.3%	4.47 (1.39–14.60)
Medication used						
Semaglutide (Ozempic®)	19.8%	1	28.3%	1	48.1%	1
Liraglutide (Saxenda®)	10.3%	0.46 (0.12–1.71)	17.2%	0.46 (0.15–1.41)	41.4%	0.82 (0.35–1.93)
Tirzepatide	30.8%	1.56 (0.84–2.91)	41.3%	1.40 (0.79–2.50)	61.5%	1.57 (0.93–2.65)
Semaglutide (Wegovy®)	30%	1.28 (0.29–5.72)	40%	0.99 (0.23–4.29)	80.0%	3.51 (0.68–18.24)
Liraglutide (Victoza®)	37.5%	2.10 (0.45–9.81)	12.5%	0.32 (0.037–2.77)	12.5%	0.17 (0.019–1.41)
Multiple medications	42.9%	3.29 (0.94–11.57)	28.6%	0.85 (0.23–3.13)	71.4%	2.03 (0.58–7.09)

## Discussion

This study adds to the limited evidence on knowledge, attitudes, and practices regarding GLP-1 receptor agonist use among users in Saudi Arabia. Overall, participants demonstrated predominantly moderate knowledge and neutral attitudes toward GLP-1 receptor agonists, whereas medication-use practices were generally more favorable, with more than half of participants reporting good practices. Significant positive associations were observed among the KAP domains, with higher knowledge levels associated with more positive attitudes and better self-reported medication-use practices. Item-level analyses revealed important gaps in users’ understanding of medication safety, adverse effects, and the role of lifestyle modification in achieving sustainable treatment outcomes. Multivariable analyses further identified older age as a predictor of lower knowledge and less positive attitudes, whereas higher educational attainment was associated with more favorable attitudes and practices. Female participants were also more likely to report good medication-use practices. These findings highlight opportunities to improve patient education and promote the safe and effective use of GLP-1 receptor agonists.

The predominance of moderate knowledge observed in this study suggests that although users are increasingly familiar with GLP-1 receptor agonists, important gaps remain regarding their safe and appropriate use. This finding is consistent with previous studies evaluating awareness of weight-loss medications and chronic disease therapies, which frequently report moderate levels of knowledge despite widespread use and extensive media exposure (Abualhommos et al., 2026; Auerbach et al., 2025). The limited awareness of serious adverse effects and monitoring requirements identified in the present study is particularly important given the growing popularity of these medications and the increasing availability of information from non-professional sources (Arillotta et al., 2023; Bremmer and Hendershot, 2024).

Participants with higher knowledge levels were more likely to demonstrate positive attitudes and favorable medication-use practices. Although the cross-sectional design does not establish the direction of these relationships, the observed associations suggest that knowledge, attitudes, and medication-use practices may be interrelated. These findings support further evaluation of educational initiatives aimed at improving users’ understanding of both the benefits and limitations of GLP-1 receptor agonists (DePietro et al., 2026; Zhou et al., 2026; Ahmad et al., 2025).

Older age was significantly associated with lower knowledge and less positive attitudes. One possible explanation is differences in exposure to digital health resources and social media platforms, although this was not established by the present study (Javaid et al., 2024; Srisaknok et al., 2025). Higher educational attainment was associated with more positive attitudes and better practices, consistent with a potential relationship between education, health literacy, and medication-related decision-making (Smith and Langdon, 2024). Female participants were also more likely to report good medication-use practices than males. The type of GLP-1 receptor agonist used was not independently associated with KAP outcomes after adjustment for the variables included in the model.

The rapid uptake of GLP-1 receptor agonists for weight management and glycemic control has created new opportunities and challenges for healthcare systems. Although participants generally reported favorable medication-use practices, the predominance of moderate knowledge and neutral attitudes indicates ongoing educational needs. Given the potential adverse effects, monitoring requirements, and increasing reports of inappropriate use, healthcare professionals should provide comprehensive counselling regarding medication administration, adverse effect management, realistic treatment expectations, and the continued importance of lifestyle modification (Mozaffarian et al., 2026; Ghusn and Hurtado, 2024).

Pharmacists may play a particularly important role in addressing these gaps because of their accessibility and frequent interactions with patients receiving GLP-1 receptor agonists (Rajiah et al., 2021). Public health campaigns and digital health initiatives may also be valuable in improving awareness and counteracting misinformation circulating through social media and other online sources (Arillotta et al., 2023). Targeted educational interventions may be especially beneficial for older adults and individuals with lower educational attainment, who were identified as potentially vulnerable groups in this study (Geboers et al., 2015). In addition, strengthening regulatory oversight of prescribing and dispensing practices in community pharmacies may help ensure appropriate access to GLP-1 receptor agonists and reduce the risk of unsupervised or inappropriate use (Alenezi et al., 2023).

The study has several strengths, including a comprehensive assessment and the use of a structured questionnaire adapted from validated KAP instruments. The questionnaire underwent expert review and pilot testing, and demonstrated good internal consistency reliability. In addition, the inclusion of participants from multiple regions of the Kingdom enhances the diversity of the study population, while multivariable analyses allowed the identification of factors independently associated with KAP outcomes.

Several limitations should be acknowledged. The cross-sectional design precludes causal inference between knowledge, attitudes, and practices (Kesmodel, 2018). Data, including medication-use practices, were self-reported and not objectively verified, introducing potential recall, social desirability, and reporting biases. Online purposive snowball sampling may have introduced selection and digital-selection biases, limited representativeness, and precluded independent verification of participant identity and eligibility. Although the achieved sample size was below the calculated target, it was comparable to or larger than those of similar studies. Residual confounding from unmeasured clinical and socioeconomic factors cannot be excluded; therefore, associations with age, sex, and education should not be interpreted as causal. Treatment indication, duration of GLP-1 receptor agonist use, and medical supervision were not specifically assessed, limiting comparisons across clinically distinct user groups. Finally, the non-probability online sampling approach limits the generalizability of the findings beyond the surveyed sample.

Future research should focus on developing and evaluating targeted educational interventions to address the knowledge gaps and misconceptions identified in this study, particularly those related to medication safety, adverse effects, monitoring requirements, and the continued importance of lifestyle modification. Given the increasing influence of social media and digital platforms on health-related decision-making, further studies are needed to examine their role in shaping perceptions and use of GLP-1 receptor agonists and to identify effective strategies for countering misinformation. In addition, intervention studies evaluating pharmacist-led counselling, multidisciplinary obesity management programs, and community-based educational initiatives are warranted to determine their effectiveness in improving knowledge, medication adherence, and safe medication-use practices. Future research should also assess the impact of regulatory and community pharmacy-based interventions, including strengthened prescription verification and dispensing practices, on promoting the appropriate use of GLP-1 receptor agonists and enhancing patient safety.

## Conclusion

GLP-1 receptor agonist users in Saudi Arabia demonstrated favorable self-reported medication-use practices but important gaps in knowledge and attitudes, particularly regarding medication safety and the role of lifestyle modification. Higher knowledge was associated with more positive attitudes and better practices, emphasizing the importance of patient education. Targeted educational interventions, pharmacist-led counselling, and public health initiatives may help promote the safe, informed, and effective use of GLP-1 receptor agonists, particularly among older adults and individuals with lower educational attainment.

## Data Availability

The original contributions presented in the study are included in the article/Supplementary Material, further inquiries can be directed to the corresponding author.
