# Supplementary material for: Knowledge, attitudes, and practices among users of GLP-1 receptor agonists in Saudi Arabia: a cross-sectional study
Source: Front Pharmacol. 2026 Oct 12;17:1912202. doi: 10.3389/fphar.2026.1912202 (PMC13697244; doi:10.3389/fphar.2026.1912202)
Supplement: Supplementary file 1 [file Supplementaryfile1.pdf]

Supplement materials

Table S1. Frequency and percentage of participants' correct knowledge regarding weight loss medications and their side effects.

Items	Correct Answer , n(%)
Modern weight loss drugs can be legally purchased without a prescription	144 (46.5)
Nausea and vomiting are common side effects	246 (78.4)
Diarrhea or constipation are common side effects	197 (63.5)
Users must inform the doctor immediately if persistent abdominal pain or repeated vomiting occurs	239 (77.1)
Modern weight loss drugs may cause Pancreatitis	124 (40.0)
Rapid weight loss while using these drugs may increase the risk of gallstones	104 (33.5)
Modern weight loss drugs may affect kidney function	185 (59.7)
Modern weight loss medications can interact with others.	138 (44.5)

Table S2. Frequency and percentage of participants' correct attitude regarding weight loss medications and their side effects.

Items	Correct Answer , n(%)
Weight loss drugs do not require a medical consultation	149 (48)
Regular follow-up with a doctor is necessary.	258 (83.2)
Drugs must be available by prescription only	200 (64.5)
Medications can replace healthy diet/exercise	74 (23.9)
Drugs are overused for cosmetic reasons only	152 (49.1)
Drugs are completely safe with no side effects	176 (56.8)
Effectiveness continues after stopping Drugs	67(21.6)
Drugs are an easy/fast way to lose weight without effort	35 (11.3)
Drugs are the best first choice for everyone	81 (26.1)

Table S3. Frequency and percentage of participants' correct practice regarding weight loss medications and their side effects.

Items	Correct Answer , n(%)
Consulting a doctor or healthcare provider before starting to use these medications	214(69)
Adhering to regular follow-up visits with the doctor	171 (55.2)
Increasing the dose gradually according to the doctor's instructions.	175(56.5)
Maintaining physical activity while using the medication.	148 (47.7)
Maintaining a healthy diet while using the medication.	147 (47.4)
Storing the modern weight loss medications in the refrigerator before use, and away from heat after opening.	252 (81.3)
Rely on social media or influencers as a primary source of information about these medications.	84 (27.1)
Buying modern weight loss medications only from licensed or certified pharmacies.	265 (85.5)
Sharing modern weight loss medications with others without a medical prescription.	64 (20.6)

Table S4. Logistic regression analysis of independent factors against knowledge of participants

Risk factors	COR (95% CI)	*P* value	aOR (95% CI)	P value
**Age**				
≤30	1		1	
31-40	0.987 (0.541–1.798)	0.965	0.884 (0.467-1.671)	0.704
41-50	0.884 (0.445–1.756)	0.725	0.931 (0.446-1.942)	0.848
More than 50	0.166 (0.016–0.279)	0.017	0.137 (0.037-0.815)	0.027
**Gender**				
Male	1		1	
Female	1.982(1.156–3.397)	0.013	1.714 (0.966-3.040)	0.065
**Education level**				
High school or less	1		1	
Diploma	0. 471(0.145–1.522)	0.208	0.422 (0.125–1.425)	0.165
Bachelor's degree	1.373(0.730–2.581)	0.326	1.151 (0.592–2.240)	0.678
Postgraduate studies	2.521(0.952–6.676)	0.063	1.953 (0.712–5.358)	0.194
**Medication Used**				
Ozempic	1		1	
Saxenda	0. 467(0.0129–1.692)	0.246	0.455 (0.121–1.713)	0.244
Mounjaro	0 1.799(0. 992– 3.262)	0.053	1.558 (0.835 – 2.907)	0.163
Wegovy	1.735(0.413– 7.280)	0.452	1.279 (0.286 – 5.721)	0.748
Victoza	2.429(0.537– 10.982)	0.249	2.100 (0.450– 9.808)	0.345
Multiple medications	3.036(0. 950– 9.696)	0.061	3.293 (0.937-11.573)	0.063

Table S5. Logistic regression analysis of independent factors against Attitude of participants

Risk factors	COR (95% CI)	*P* value	aOR (95% CI)	P value
**Age**				
≤30	1		1	
31-40	1.224(0.704–2.130)	0.474	1.092(0.600–1.988)	0.773
41-50	0.503 (0.251–1.008)	0.053	0.523 (0.247–1.107)	0.090
More than 50	0.241 (0.080–0.729)	0.012	0.289 (0.092–0.911)	0.034
**Gender**				
Male	1			
Female	2.2021(1.231–3.317)	0.005	1.649(0.963-2.823)	0.068
**Education level**				
High school or less	1		1	
Diploma	0.850(0.317–2.275)	0.746	0. 819(0.296–2.268)	0.702
Bachelor's degree	1.754(0.951–3.236)	0.072	1. 687(0.887–3.208)	0.111
Postgraduate studies	7.960(2.852–22.215)	<0.001	6. 507(2.230–18.988)	0.001
**Medication Used**				
Ozempic	1		1	
Saxenda	0.528(0.184–1.511)	0.234	0.461 (0.151-1.408)	0.174
Mounjaro	1.779(1.039– 3.047)	0.036	1.401 (0.786-2.498)	0.253
Wegovy	1.689(0. 445– 6.411)	0.441	0.987 (0.227-4.286)	0.986
Victoza	0.362 (0.0 43– 3.068)	0.351	0.319 (0.037-2.771)	0.300
Multiple medications	1.013(0. 295– 3.481)	0.983	0.849 (0.231-3.128)	0.806

Table S6. Logistic regression analysis of independent factors against practices of participants

Risk factors	COR (95% CI)	*P* value	aOR (95% CI)	P value
**Age**				
≤30	1			
31-40	0.825(0.478–1.424)	0.491		
41-50	0. 697(0.378–1.285)	0.247		
More than 50	0.926(0.424–2.022)	0.846		
**Gender**				
Male	1		1	
Female	2.293(1.448–3.630)	<0.001	2.063(1.279-3.329)	0.003
**Education level**				
High school or less	1		1	
Diploma	1. 281(0.571–2.876)	0.732	1.234(0.536–2.842)	0.621
Bachelor's degree	1. 384(0.808–2.369)	0.199	1.348(0.767–2.369)	0.299
Postgraduate studies	5.694(1.780–18.222)	0.003	4.469(1.368–14.602)	0.013
**Medication Used**				
Ozempic	1		1	
Saxenda	0.761(0.331–1.748)	0.520	0.815(0.345-1.925)	0.641
Mounjaro	1. 725(1. 038– 2.869)	0.036	1.565 (0.925-2.648)	0.095
Wegovy	4. 314(0.875– 21.273)	0.073	3.518 (0.678-18.239)	0.134
Victoza	0.1 54(0.018 – 1.296)	0.085	0.165 (0.019-1.414)	0.100
Multiple medications	2. 696(0.796-9.137)	0.111	2.032 (0.583-7.088)	0.266
